# Treatment Discontinuation and Clinical Events in Type 2 Diabetes Patients Treated with Dipeptidyl Peptidase-4 Inhibitors or NPH Insulin as Third-Line Therapy

**DOI:** 10.1155/2018/4817178

**Published:** 2018-03-11

**Authors:** Cristiano S. Moura, Zale B. Rosenberg, Michal Abrahamowicz, Sasha Bernatsky, Hassan Behlouli, Louise Pilote

**Affiliations:** ^1^Centre for Outcome Research & Evaluation (CORE), McGill University, Montréal, QC, Canada H3A 0G4; ^2^Department of Epidemiology, McGill University, Biostatistics and Occupational Health, Montréal, QC, Canada H3A 0G4; ^3^Division of General Internal Medicine, McGill University, McGill University Health Centre, Montréal, QC, Canada H3A 0G4

## Abstract

**Objective:**

To compare dipeptidyl peptidase-4 (DPP-4) inhibitors with neutral protamine Hagedorn (NPH) insulin, in terms of effectiveness and safety for the management of patients with type 2 diabetes mellitus (DM2) not controlled on metformin and sulfonylureas.

**Methods:**

A retrospective cohort study of individuals with DM2 newly dispensed with either DPP-4 inhibitors or NPH as third-line therapy, after metformin and sulfonylurea. Treatment discontinuation, macrovascular outcomes, and hypoglycemia were compared using multivariable Cox regression models, adjusted for sex, age, year of cohort entry, place of residence, hypertension, past history of hypoglycemia, diabetic ketoacidosis, comorbidities, and number of visits to emergency departments, outpatient physician, and hospitalizations.

**Results:**

Treatment discontinuation and hypoglycemia occurred more frequently with NPH than with DPP-4 inhibitor users. In the adjusted Cox model, the use of NPH compared to that of DPP-4 inhibitors was associated with a higher risk of discontinuation (HR: 1.33; 95% CI 1.27–1.40) and hypoglycemia (HR: 2.98; 95% CI 2.72–3.28). Risk of cardiovascular events was similar across groups.

**Conclusions:**

This real-world analysis suggests that DM2 patients initiating third-line therapy with NPH have poorer control of diabetes when compared to DPP-4 inhibitor initiators.

## 1. Introduction

Between 7% and 9% of North Americans have type 2 diabetes mellitus [1, 2], often requiring medication [3]. Most guidelines suggest metformin as initial therapy, but there is uncertainty as to which medications should be added when metformin is insufficient [3, 4]. Sulfonylureas are commonly used as second-line therapy. In addition, dipeptidyl peptidase-4 (DPP-4) inhibitors and neutral protamine Hagedorn (NPH) insulin are both effective add-on therapies. However, there are few data directly comparing these two options. According to one recent cost-effectiveness analysis, the addition of NPH insulin to metformin and sulfonylurea combination therapy is the most cost-effective strategy; however, the use of a DPP-4 inhibitor is potentially cost-effective when higher rates of hypoglycemia are assumed [5]. More recently, the Canadian Diabetes Association Clinical Practice Guideline has recommended to add sodium-glucose cotransporter 2 (SGLT2) inhibitors, such as empagliflozin, in patients with type 2 diabetes with clinical cardiovascular disease [6]. Despite the variety of treatment options, reaching target glucose levels remains a challenge for many patients with type 2 diabetes mellitus [7, 8]. In this study, we sought to compare DPP-4 inhibitors with intermediate-acting NPH insulin in terms of effectiveness and safety for the management of patients with type 2 diabetes mellitus not controlled on metformin and sulfonylureas.

## 2. Methods

### 2.1. Data Source

For this study, we used a retrospective cohort design with data from the MarketScan Commercial Claims and Encounters Database from January 1, 2010, to December 31, 2014. This United States (US) administrative database contains claim data for millions of privately insured patients with many different health plans from large employers, public organizations, and government. This data encompasses demographic information, enrollment details, ICD-9-CM (International Classification of Diseases, 9th revision, clinical modification) codes from inpatient and outpatient healthcare encounters, and pharmacy claims from outpatient pharmacies.

### 2.2. Study Population

We studied patients with type 2 diabetes mellitus newly dispensed with either NPH insulin or a DPP-4 inhibitor as third-line therapy between January 2011 and December 2014. Patients were deemed to be third-line initiators if they filled prescriptions for both metformin and sulfonylurea in the 90 days prior to cohort entry. The date of the first prescription of the third-line agent was defined as the date of cohort entry, and a six-month preperiod was used to establish new users. We identified type 2 diabetes mellitus patients as those with at least one outpatient or inpatient claim with specific ICD-9-CM codes for type 2 diabetes mellitus (250.X0 or 250.X2) or a mix of both type 2 and unspecific diabetes mellitus diagnoses (250.x) with no code for type 1 diabetes (250.x1/250.x3) at any point before cohort entry or one month after [9]. We further excluded patients with claims containing an ICD-9-CM code for gestational diabetes (648.8x). The analysis was restricted to individuals who were covered for medical and pharmacy benefits from their plan during the preperiod (six months before cohort entry).

### 2.3. Exposure Assessment

Patients were classified as either NPH insulin or DPP-4 inhibitor initiators, according to the medication dispensed at cohort entry. The number of supply days was ascertained from the prescription database and used to calculate duration of exposure for each drug. Individuals were assumed to be continuously exposed from the date of prescription to the end of supply days. Overlapping periods between prescriptions, in case of early refill, were disregarded. Instead, we included a maximum permissible gap of 90 days between refills to account for any remaining stockpiled medications.

### 2.4. Outcomes

The primary outcome was the time to treatment discontinuation, defined as no claim for the same index drug in the 90 days after exhausting the supply provided in the most recent prescription. A 90-day gap-based measure has been used in previous studies evaluating the persistence of insulin regimens in type 2 diabetes mellitus patients [10, 11]. This analysis was restricted to individuals initiating their treatment before October 1, 2014, to allow the assessment of the 90-day gap for all individuals. In a separate analysis, cardiovascular events and hypoglycemia were examined. Cardiovascular events were defined as a composite of acute myocardial infarction (ICD-9-CM codes: 410.xx), unstable angina (411.1x), coronary artery bypass graft (36.10–36.19 and Current Procedural Terminology-4 [CPT-4] codes 33510–33519, 33521–33523, and 33533–33536), coronary revascularization or percutaneous coronary intervention (00.66 and 36.01-36.09 plus CPT-4 codes 92980–92982, 92984, 92995, and 92996). Hypoglycemic events were identified using primary and nonprimary ICD-9-CM diagnosis codes (250.8, 251.0–251.2, and 962.3) recorded on outpatient and inpatient services.

### 2.5. Covariates

A priori, we selected and controlled for potential confounders of association between the drugs of interest and outcomes. These included sex, age at cohort entry, year of cohort entry, and place of residence (urban or rural area). Other covariates, based on the year prior to enrollment, included the number of visits to emergency departments, the number of outpatient physician visits (allowing one per physician per day), the number of hospitalizations, prior diabetic ketoacidosis (250.1x), hypoglycemia, and the Deyo-Charlson comorbidity index [12]. The comorbidity index was calculated using ICD-9-CM codes (excluding codes for diabetes and diabetes with chronic complications) and required one or more hospitalization or more than one outpatient claim with that comorbidity.

### 2.6. Statistical Analysis

Baseline characteristics of the study population were described using frequency distributions for categorical variables, as well as means, medians, and interquartile ranges (IQR) for continuous variables. Incidence rates (number of events per 100 person-years), with 95% confidence intervals (95% CI) of discontinuation; hypoglycemia; and cardiovascular events were estimated, separately for NPH and DPP-4 initiators.

Time-to-event methods for right-censored data were used to compare the risks of each specific outcome between patients who initiated their treatment with NPH insulin and those who initiated their treatment with DPP-4. The cumulative incidence of discontinuation was assessed using the Kaplan-Meier method. We estimated the adjusted hazard ratios (HR) with 95% CI using Cox proportional hazards models to assess, in separate analyses, the risks of (i) discontinuation, (ii) cardiovascular outcomes, and (iii) hypoglycemia. In all models, the DPP-4 inhibitor was used as the reference group. The models were adjusted for the variables listed in Section 2.5. In all analyses, patients were followed up from cohort entry (time 0) to the first occurrence of the outcome of interest or the earliest one of the following censoring events: loss of medical or pharmacy coverage, inpatient death, or reaching the study end date of December 31, 2014. In the analyses that focused on clinical outcomes and hypoglycemia, patients were additionally censored at the time of their discontinuation of the initial treatment. Similar to other MarketScan-based studies, information on deaths occurring outside of the inpatient settings was not available in the database [13, 14]. The proportional hazards assumption was verified by testing the significance of interactions between the covariates and follow-up time. This method indicated that the assumption was violated for a binary indicator of “prior hypoglycemia.” We therefore stratified this variable in the final multivariable Cox model with hypoglycemia as an outcome. To explore if the effect of the initial treatment differed between women and men, we tested two-way sex-by-drug interactions. Statistical significance in all analyses was tested with the model-based Wald test at 2-tailed *α* = 0.05. All analyses were conducted using the SAS version 9.4.

## 3. Results

**Figure 1:**
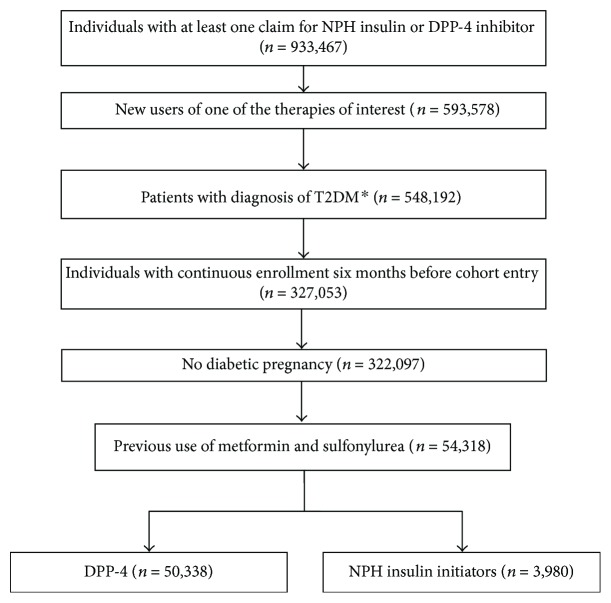
Flowchart of cohort selection. ^∗^Individuals with outpatient or inpatient claim with specific T2DM ICD-9-CM codes (250.x0/250.x2) or a mix of claims with type 2 and unspecified DM diagnosis (code 250.x) at any point before cohort entry or one month after the first prescription. Individuals with type 1 DM (250.x1/250.x3) codes at any point before cohort entry were excluded.

Among a total of 933,467 individuals who filled at least one prescription of the medications of interest, 54,318 met the inclusion and exclusion criteria and were included in the analysis (Figure 1). Of these, 50,338 (92.7%) filled prescriptions for DPP-4 inhibitor medications, while 3980 (7.3%) filled prescriptions for NPH insulin. The median supply days was 30 days (IQR: 28–86 days) for NPH and 30 days (IQR: 30–30 days) for DPP-4. The clinical characteristics of DPP-4 inhibitor and NPH insulin initiators are shown in Table 1. The DPP-4 inhibitor group included slightly more women (59.5% versus 52.8% in the NPH insulin group). Patients in the NPH insulin group had more hypoglycemia in the year before cohort entry (21.0% versus 4.1%) and higher frequency of one or more comorbidity (Table 1).

### 3.1. Treatment Discontinuation

**Figure 2:**
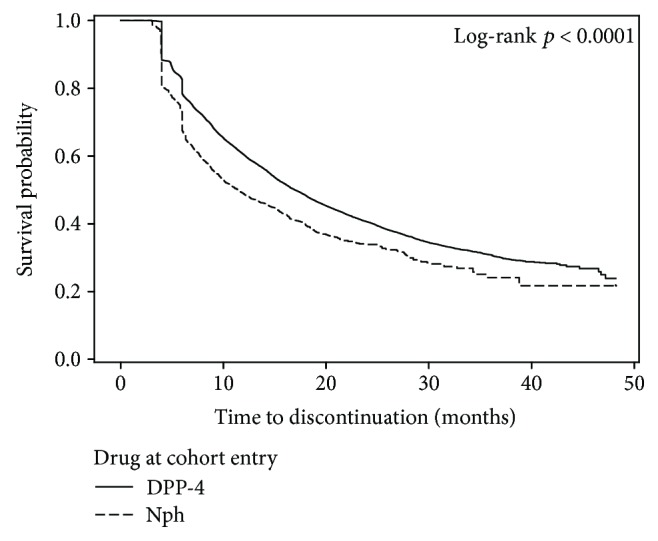
Kaplan-Meier curves for discontinuation of therapy (prescription gap > 90 days) in type 2 diabetes mellitus patients initiating NPH insulin or DPP-4 inhibitors, 2011–2014.

During follow-up, treatment discontinuation occurred more frequently among NPH insulin users than among DPP-4 inhibitor users (Figure 2), and this difference remained statistically significant in the multivariable Cox model (adjusted HR = 1.33; 95% CI: 1.27–1.40; Table 2).

### 3.2. Hypoglycemia and Cardiovascular Events

Overall, 2887 patients experienced at least one event of hypoglycemia during follow-up; the unadjusted rate among NPH users was 28.6/100 person-years (95% CI: 26.5–30.8), and that among DPP-4 inhibitor users was 5.1/100 person-years (95% CI: 4.9–5.3) (Table 3). The multivariable results confirmed a statistically very significant almost threefold increase in the risk of hypoglycemia among NPH insulin initiators compared to DPP-4 inhibitor initiators (adjusted HR = 2.82; 95% CI: 2.57–3.10; Table 2).

During the follow-up, 1083 patients had at least one cardiovascular event (CVD); the CVD rates did not differ materially between the two treatment groups: 2.31/100 person-years (95% CI: 2.17–2.46) among NPH users versus 2.44/100 person-years (95% CI: 1.92–3.08) among DPP-4 inhibitor users. The multivariable Cox model confirmed that the risk of cardiovascular outcomes was very similar across the two groups (adjusted HR = 1.08; 95% CI: 0.84–1.39). There were no significant interactions between sex and initial treatment in the analyses with hypoglycemia or cardiovascular events as outcomes.

## 4. Discussion

We found significantly less treatment discontinuation and lower risk of hypoglycemia in patients with type 2 diabetes newly dispensed with DPP-4 inhibitors compared to those initiating NPH insulin, when these agents were used as third-line therapy after metformin and sulfonylurea. To our knowledge, this was the first observational study to compare treatment discontinuation and clinical outcomes between initiators of these medications in a type 2 diabetes population.

Examining discontinuation is particularly relevant in diabetes, a chronic condition that requires long-term glycemic control to prevent complications [15]. The discontinuation rate among DDP-4 inhibitor initiators found in our study was similar to that reported by Farr et al. [16] after 1-year follow-up and consistent with that reported by Rathmann et al. [17]. Possible factors influencing therapy discontinuation in type 2 diabetes mellitus include complexity of dosing, perception of efficacy, and adverse events. In addition, a patient's resistance to an injection regimen and fear of hypoglycemia could explain the particularly high rate of discontinuation among insulin initiators [18, 19]. A high rate of discontinuation was reported in the study conducted by Bonafede et al. ([20] on type 2 diabetes mellitus patients: in one-year follow-up, 75% of basal insulin initiators and 65% of insulin mixture initiators had a discontinuation period, defined as a greater than 90-day gap in their insulin prescriptions. Ascher-Svanum et al. [21] studying discontinuation and factors associated in a type 2 diabetes mellitus population reported high probability of early discontinuation for both basal insulin users and insulin mixture users.

Hypoglycemia is a common and potentially dangerous adverse event [22]. Some patients with previous hypoglycemia episodes were prescribed NPH insulin, which seems to be contradictory since this medication would not be the preferable treatment for patients with recurrent hypoglycemia episodes [23]. On the other hand, in difficult-to-control patients, medications that increase the risk of hypoglycemia, including insulin, may be needed. Progressive insulin deficiency is also associated with a higher risk of hypoglycemia [24]. The risk of developing a hypoglycemia episode after cohort entry was also higher among NPH insulin initiators The rate of events in the DPP-4 inhibitor group was similar to that found in a pooled analysis of 19 double-blind clinical studies with type 2 diabetes patients taking sitagliptin or comparator agent (placebo or an active comparator) [25]. To date, there is no head-to-head study between DPP-4 inhibitor and NPH insulin to compare these events. A recent network meta-analysis found no significant differences in severe hypoglycemia between DPP-4 inhibitors and placebo used as third-line therapy [26]. In a retrospective analysis comparing several oral antidiabetic medications, Bron et al. [27] reported a significantly decreased risk of hypoglycemia associated with the use of DPP-4 inhibitors and an increased risk of hypoglycemic events among users of insulin, sulfonylureas, and/or other oral diabetes medications (meglitinide and *α*-glucosidase inhibitors).

In our study, the risk for cardiovascular outcomes was similar between patients using DPP-4 inhibitors and those using NPH insulin, which is consistent with prior trials. Zannad et al. [28] found that alogliptin did not increase the risk of heart failure outcomes in the EXAMINE trial. Also, the SAVOR-TIMI 53 trial [29] found that the secondary endpoint of a composite of cardiovascular death, myocardial infarction, stroke, hospitalization for unstable angina, coronary revascularization, or heart failure was similar between patients in the saxagliptin group and patients in the placebo group, but more patients in the saxagliptin group were admitted to hospital for heart failure. In a population-based cohort study, Wang et al [30] found similar risks of hospitalization for heart failure and percutaneous coronary intervention between sitagliptin group and nonsitagliptin group.

### 4.1. Limitations

Claim data are not collected for research purposes, and consequently, some misclassification can occur. Important clinical, demographic, and lifestyle-related risk factors for diabetes were lacking on the database. Baseline information on hemoglobin A_1C_ and BMI was available for a small subsample (less than 5%). Because of that, we were unable to assess whether discontinuation is related to failure to achieve glycemic control or not. As with all retrospective observational studies, we cannot establish causality. Treatment discontinuation may actually have been a result of improved glycemic control. For example, patients in one treatment group may have been started on a third-line agent due to a factor that temporarily worsened diabetic control (such as concomitant prednisone use), and it may be that eventually glycemic control normalized and the third-line drug was stopped. Finally, when using data from a pharmacy database, it is not possible to verify if the drug was actually taken as prescribed.

In conclusion, patients initiating DPP-4 inhibitors as third-line therapy in type 2 diabetes appeared to have lower discontinuation and have less hypoglycemia when compared to patients starting NPH insulin. These findings support previous evidence suggesting that DPP-4 inhibitor agents are well tolerated in type 2 diabetes mellitus patients who have previously failed with metformin plus sulfonylurea.

## Figures and Tables

**Table 1:** Baseline characteristics of type 2 diabetes mellitus patients with new users of NPH insulin or DPP-4 inhibitors, MarketScan database, 2011–2014.

Characteristics	DPP-4 inhibitor (*n* = 50338)	NPH insulin (*n* = 3980)
Female sex, *n* (%)	29,961 (59.5)	2103 (52.8)
Age in years, median (IQR)	58 (51–64)	57 (50–63)
Urban residency, *n* (%)^∗^	40,336 (82.3)	3511 (89.6)
Year of cohort entry (%)		
2011	5196 (10.3)	293 (7.4)
2012	21,234 (42.2)	1714 (43.1)
2013	12,597 (25.0)	1276 (32.1)
2014	11,311 (22.5)	697 (17.5)
Comorbidities		
Hypoglycemia, *n* (%)^†^	2040 (4.1)	836 (21.0)
Dyslipidemia, *n* (%)^†^	33,992 (67.5)	2473 (62.1)
One or more comorbidity, *n* (%)^†^	4647 (9.2)	471 (11.8)
Coronary artery disease, *n* (%)^‡^	1489 (3.0)	114 (2.9)
Number of hospitalizations, mean (SD)^†^	0.12 (0.41)	0.19 (0.61)
Number of physician visits, mean (SD)^†^	10.2 (10.3)	9.9 (11.5)
Number of ED visits, mean (SD)^†^	0.33 (0.93)	0.50 (1.25)

SD: standard deviation; IQR: interquartile range; CCI: Charlson comorbidity index; ED: emergency department. ^∗^Unknown residence status = 1403. ^†^Based on outpatient and inpatient claims in the year preceding cohort entry. ^‡^ICD-9-CM code for acute myocardial infarction, percutaneous coronary intervention, coronary revascularization, unstable angina, or coronary artery bypass grafting in the preceding year.

**Table 2:** Results of Cox regression analysis for treatment discontinuation, cardiovascular events, and hypoglycemia with NPH insulin, MarketScan, 2011–2014.

Outcome	Unadjusted		Adjusted^∗^	
	HR^∗∗^	95% CI	HR^∗∗^	95% CI
Treatment discontinuation	1.41	1.34–1.48	1.33	1.27–1.40
Cardiovascular events	1.02	0.80–1.30	1.08	0.84–1.39
Hypoglycemia^∗∗∗^	5.22	4.79–5.69	2.82	2.57–3.10

HR: hazard ratio; CI: confidence interval. ^∗^All models were adjusted for sex, age at cohort entry, year of cohort entry, place of residence, and the following one-year prior to enrollment variables: comorbidities, number of visits to emergency departments, number of outpatient visits, number of hospitalizations, diabetic ketoacidosis, and hypoglycemia. ^∗∗^DPP-4 is the reference category. ^∗∗∗^The model with hypoglycemia as an outcome was stratified on the previous occurrence of hypoglycemia.

**Table 3:** Number of cases, person-years, and rates per 100 person-years with 95% CI of hypoglycemia (overall and stratified by the previous episode of hypoglycemia) and cardiovascular events in type 2 diabetes mellitus patients initiating NPH insulin or DPP-4, 2011–2013.

Group	Number of events	Time to event (years), median (IQR)	Time at risk (years)	Rate (per 100 person-years)	95% CI
Hypoglycemia (overall)					
NPH	694	0.41 (0.25–0.77)	2430	28.6	26.5–30.8
DPP-4	2193	0.60 (0.33–1.19)	43,139	5.1	4.9–5.3
Hypoglycemia (with previous hypoglycemia episode^∗^)					
NPH	441	0.27 (0.10–0.49)	301	146.4	133.3–160.7
DPP-4	620	0.37 (0.17–0.81)	1206	51.4	47.5–55.6
Hypoglycemia (without previous hypoglycemia episode)					
NPH	253	0.48 (0.32–0.87)	2129	11.9	10.5–13.4
DPP-4	1573	0.61 (0.33–1.21)	41,933	3.8	3.6–3.9
Cardiovascular events					
NPH	1014	0.49 (0.32–0.95)	43,867	2.31	2.17–2.46
DPP-4	69	0.60 (0.32–1.21)	2833	2.44	1.92–3.08

^∗^One year before cohort entry. IQR: interquartile range (Q1–Q3).
